# Gasdermin D: A potential mediator and prognostic marker of bladder cancer

**DOI:** 10.3389/fmolb.2022.972087

**Published:** 2022-09-01

**Authors:** Randa El-Gamal, Mona Abdelrahim, Mohamed El-Sherbiny, Eman T. Enan, Mohammad El-Nablaway

**Affiliations:** ^1^ Department of Medical Biochemistry, Faculty of Medicine, Mansoura University, Mansoura, Egypt; ^2^ Medical Experimental Research Center (MERC), Faculty of Medicine, Mansoura University, Mansoura, Egypt; ^3^ Consultant of Pathology, Urology and Nephrology Center, Mansoura University, Mansoura, Egypt; ^4^ Department of Basic Medical Sciences, Anatomy Unit, College of Medicine, AlMaarefa University, Riyadh, Saudi Arabia; ^5^ Department of Pathology, Faculty of Medicine, Mansoura University, Mansoura, Egypt; ^6^ Medical Biochemistry Unit, Department of Basic Medical Sciences, College of Medicine, Almaarefa University, Riyad, Saudi Arabia

**Keywords:** bladder cancer, gasdermin D, expression, prognostic marker, tumor recurrence

## Abstract

**Background:** Bladder cancer is considered one of the commonest widespread cancers, its presentation ranges from non-muscle invasive form to being muscle-invasive. The gasdermin family of proteins consists of six proteins. Members of gasdermin family are involved in pyroptosis; which is considered as type of inflammatory apoptosis via participation of gasdermin D and inflammatory caspases.

**Purpose:** The goal of this research was to look into the potential involvement of gasdermin D in pathogenesis of bladder cancer, In addition, to investigate its potential role as a prognostic marker of bladder cancer.

**Methods:** Gasdermin D gene and protein expression was examined in fresh frozen 80 bladder cancer specimens (30 NMIBC, and 50 MIBC) and the matching 80 control tissue samples utilizing real-time polymerase chain reaction and western blotting. Furthermore, the immunoreactivity of gasdermin D protein was also detected by immunohistochemistry.

**Results:** Gasdermin D gene and protein expression showed a highly significant difference between the control and the two bladder cancer groups (*p* < 0.001), as demonstrated by real-time PCR, western blotting and immunohistochemistry. Cox proportional hazards regression models showed that lower gasdermin D gene expression in cancer patients (≤1.58-fold), and younger age (≤53 years) were linked with a higher risk of local tumor recurrence. Moreover, higher gasdermin D gene expression (>2.18-fold), and lymph nodes’ involvement were associated with an increased mortality.

**Conclusion:** Gasdermin D is involved in the pathogenesis of bladder cancer and muscle invasion, in addition, tissue gasdermin D expression may be used as useful tool to predict local tumor recurrence.

## 1 Introduction

Bladder cancer (BC) is the fourth most prevalent cancer in adult males (Lenis et al., 2020). About 549,393 new cases of BC was reported in 2018 (Torre and Jemal, 2018). Every year, about 170,000 deaths from BC worldwide (Tran et al., 2021). It can present as a non-invasive, unaggressive tumor with long-term surveillance or as an aggressive, invasive tumor with high mortality rates (Lenis et al., 2020). It is divided into three types: non-muscle invasive bladder cancer (NMIBC), muscle invasive bladder cancer (MIBC), and metastatic bladder cancer (Patel et al., 2020)*.*

Genetic factors, age, sex, cigarette smoking, chemical and radiation exposure and intake of certain drugs have been implicated in the initiation and progress of BC. Other environmental factors, such as diet, nutrition, and metabolic syndrome, may play a role (Fankhauser and Mostafid, 2018). Many malignancies are affected by inflammation, which is a major trigger for their development and progression (Hayashi et al., 2020).

The physiological function of bladder is directly affected by cystitis. It can occur as a result of both infectious and non-infectious agents. Gram-negative bacteria like *Proteus* and *Pseudomonas*, as well as Gram-positive bacteria like *Staphylococcus* saprophyticus, and group B streptococci, can all cause infections. Nevertheless, the most prevalent cause of infectious cystitis is *Escherichia coli* (Cui et al., 2019). Non-infectious cystitis can occur as a result of certain drugs intake, chemical and radiation exposure, or it can be idiopathic, as interstitial cystitis; other conditions, like gynecological malignant tumors, pelvic inflammatory diseases, and Crohn’s disease, can be correlated with it (Chen et al., 2020)*.*

Chronic inflammation can induce DNA damage via creation of reactive oxygen and nitrogen species (Ten Doesschate et al., 2019). These reactive species such as superoxide anion, hydrogen peroxide, and nitric oxide are produced by cells involved in inflammation like neutrophils, monocytes, and macrophages (Ambite et al., 2016). By activating certain signal pathways, reactive species can cause single or double-stranded DNA breaks, and modification of RNA, lipid, and protein resulting in tumor promotion (Fusco et al., 2020). In addition, chronic inflammation activates inducible nitric oxide synthase resulting in the production of large quantities of nitric oxide which inhibits DNA repair and stimulates angiogenesis (Oleson and Corbett, 2018).

Inducible nitric oxide synthase has been expressed only in transitional cell carcinoma of the bladder when compared with normal bladders. In addition, the density of microvessels was significantly higher in tumors that tested positive for inducible nitric oxide (Fahey and Girotti, 2015).

Gasdermins (GSDM) are a gene family that includes both the GSDM gene family and genes that are related to the GSDM. Because they were discovered to be primarily expressed in the gastrointestinal tract and skin, GSDMs were named after the terms “gastro” and “dermato.”. Despite the fact that GSDM family members are broadly expressed and may have tissue-specific activities, their functions that are content-dependent are unknown. The GSDM family members appear to be involved in a variety of physiologic and pathological activities as cellular differentiation, proliferation, apoptosis, mitochondrial homeostasis, inflammatory response, and carcinogenesis (Zou et al., 2021).

Gasdermin A-E and deafness autosomal recessive type 59 (DFNB59) are the six proteins that comprise the gasdermin protein family. With the exception of DFNB59, the gasdermin family plays a pivotal role in pyroptosis (Feng et al., 2018).

Gasdermin D (GSDMD) is a protein containing a peptide chain connecting its 31 kDa N-terminus (GSDMD-N) and 22 kDa C-terminus (GSDMD-C), is encoded by the GSDMD gene on chromosome 8 (8q24.3). When activated, the linker peptide chain is broken, separating GSDMD -N from its autoinhibitory domain (GSDMD-C) (de Vasconcelos et al., 2019). GSDMD-N forms a transmembrane opening through which cellular cytokines like IL-1b (Evavold et al., 2018) and IL-18 (Xiao et al., 2018) are released, as well as disrupting ion and water regulation (Banerjee et al., 2018), eventually leading to pyroptosis.

Pyroptosis is a type of inflammatory apoptosis distinguished by the major modulator of pyroptotic cell death GSDMD and inflammatory caspases activity (de Vasconcelos et al., 2019). Inflammatory caspases comprise caspases 1, 4, and 5 in Human and caspases 1 and 11 in Murinae (Aglietti et al., 2016). Pyroptosis’ primary function is to elicit powerful inflammatory reactions that contribute in immune response against micro-organism infection (Burdettea et al., 2021). The pyroptotic pathways are significant therapeutic targets as they are involved in pathogenesis of various illness such as sepsis (Wu et al., 2019), Alzheimer’s disease (Flores et al., 2018), HIV infection (Doitsh et al., 2014), and gout (Szekanecz et al., 2019).

GSDMD is a 53-kDa protein located downstream of the pyroptotic caspases (Runkel et al., 2004). GSDMD was discovered as a major modulator of pyroptotic cell death mediated by caspase-1 and caspase-11 downstream in several important research published in 2015 (Kayagaki et al., 2015). GSDMD has been implicated in the etiology and severity of bladder cancer in a few publications.

Peng et al. (2020) have revealed that GSDMD gene expression was higher in BC samples than in control samples, and BC patients with CD147 overexpression had a link between GSDMD and a dramatically worse prognosis and overall survival rate, suggesting that GSDMD may act as an unfavorable prognostic marker.

Besides that, GSDM family members GSDMA-C, DFNA5 have been linked to a possible involvement in a variety of cancers. GSDMD protein levels were associated with a significant increase in non-small cell lung cancer (Gao et al., 2018). Primary esophageal squamous cell carcinoma and gastric cancer cells showed low expression of GSDMA, GSDMC, and GSDMD, also, GSDME expression was ameliorated in breast, gastric, and colorectal cancers (Rogers et al., 2019). Till now, only one publication demonstrated that GSDMD may be implicated in the etiology and severity of bladder cancer (Peng et al., 2020).

### 1.1 Aim of the work

This study aimed to investigate GSDMD gene expression in bladder cancer tissue samples in comparison to control samples to assess its potential role in pathogenesis and aggressiveness of bladder cancer, thus the role of GSDMD as a new possible target for management of bladder cancer. In addition, the study aimed to investigate its possible role as a prognostic marker of bladder cancer.

## 2 Subjects and methods

### 2.1 Sample size

Consistent with a past study (Peng et al., 2020), the authors hypothesized at least a medium effect size (f = 0.25) for GSDMD gene and protein expression between the three study groups. In a one-way ANOVA study, the required sample sizes were 80 for control group, 30 for non-muscle invasive BC (NMIBC) group, and 50 for muscle invasive BC (MIBC) group. Using an F test with a 0.0500 significance value, the total sample of 160 patients attains 81 percent power to distinguish differences between the means as opposed to the alternative of equal means. We represent the size of the means variation by the effect size f = σm/σ, which is 0.2500. Calculation of sample size was performed by PASS 15 Power Analysis and Sample Size Software (2017). NCSS, LLC. Kaysville, Utah, United States, ncss. com/software/pass.

### 2.2 Samples collection

Eighty tissue samples were obtained from frozen sections of patients with BC who were pathologically diagnosed at Mansoura Urology and Nephrology Center from July 2015 to March 2016. In addition, as a control group, 80 samples from frozen sections of nearby control tissues that had been pathologically proven as being clear of BC. The ages of all patients ranged from 35 to 75. The University of Mansoura’s Faculty of Medicine’s Institutional Review Board Committee [Code number: R.21.07.1373] approved the research protocol. After 60 months of sampling, data was collected and all patients were followed up on for survival time by referring to their medical records and calling them.

Patients who had received neoadjuvant chemotherapy or radiotherapy, as well as those with acute or chronic kidney injury, endocrine problems, other malignancies, and upper urinary tract illnesses, were all excluded from the study.

The American Joint Committee on Cancer (AJCC) (2002) classified bladder cancer samples into two groups: **group A:** non-muscle invasive BC (NMIBC), which included stages 0 and I, and **group B:** muscle invasive BC (MIBC), which included stages II, III, and IV. Patients who received neoadjuvant chemotherapy or radiotherapy, as well as those with other cancers or liver disease, were excluded from the study.

### 2.3 Detection of gasdermin D mRNA expression by real-time PCR

Five strokes of liquid nitrogen were used to homogenize tissue samples. The whole cellular RNA extraction was performed utilizing QIAzol reagent (Catalog no. 79306, Qiagen, Germany) in accordance with the rules provided by the company. The RNA concentration was detected utilizing the Thermo Scientific NanoDrop 2000. (United States). The Bioline cDNA synthesis kit was used to reverse transcribe 1ug of RNA (Catalog no. BIO-65053, Bioline, United States).

A total of 20 ul was used in quantitative real-time PCR (qRT-PCR) [10 μl of HERA SYBR green PCR Master Mix (Catalog no. WF1030400X, Willowfort, United Kingdom), 2 μl of cDNA template, 2 μl (10 pmol/μl) of each gene primer and 6 μl of nuclease-free water] utilizing a real-time PCR thermocycler (Pikoreal 96, ThermoScientific, United States) The thermal profile was set at 95°C for 2 min, followed by 40 cycles of denaturation at 95°C for 10 s, then annealing and extension at 60°C for 30 s. The sequences of the primer pairs that were employed were: GSDMD forward, 5′ CGT​TCG​GGG​TGA​TGA​TTG​AA 3′, reverse, 5′ TCC​TGG​GTT​CTA​GCA​GCA​AA 3’ (RefSeq: NM_001166237.1) and the size of PCR yield was 187 bp, β-actin forward, 5′ GTG​GCC​GAG​GAC​TTT​GAT​TG 3′, reverse, 5′ GTG​GGG​TGG​CTT​TTA​GGA​TG 3’ (RefSeq: NM_001,101.4) and the size of PCR yield was 104 bp, As a control gene, the β-actin gene was utilized. Using Primer 3 software (v.4.1.0) (Primer3 Input, 2022), the primer sets were assigned, and Primer- BLAST program was utilized to assess the specificity of primer sets (Primer designing tool, 2022). To determine the specificity of the PCR products, a melting curve analysis was performed. Vivantis provided the primer sets (Vivantis Technologies, Malaysia). The following equation ΔCt = Ct gasdermin D—Ct control gene was used to represent relative expression levels; fold change of gene expression was estimated in accordance with the 2^−ΔΔCT^ method (Livak and Schmittgen, 2001).

### 2.4 Detection of gasdermin D protein in tissue samples by western blotting

Utilizing the QIAzol reagent (Catalog no. 79306, Qiagen, Germany), the total protein extraction from tissues was performed, then the protein concentration was detected using the Bradford method (Catalog no. AR0145, Bosterbio, Canada). SDS-PAGE (10%) was utilized to separate equal amounts of proteins (20 μg) and a pre-stained protein molecular weight marker (Catalog no. 161–0305, Bio-Rad, United States) (Gallaghar et al., 1989). After transferring to 0.22 mm nitrocellulose membrane (catalog no. ab133413, Abcam, United States) utilizing Eco-Line Biometra apparatus (Gottingen, Germany), incubation in 5% non-fat milk (a blocking agent) for 1 hour at 37°C was performed. Antibodies against GSDMD (catalog no. sc-393581, dilution 1: 500) and β-actin (catalog no. ab227387as a control protein) were incubated overnight at 4°C on the membranes (Abcam ab227387, dilution 1: 5000) (Hames and Rickwood, 1990). After incubation with the secondary antibody, bound proteins were visualized using chemiluminescence detection kit (Thermoscientific, United States). The chemiluminescent substrate (ClarityTM WesternECL substrate, Catalog no. 1705061, Bio-Rad, United States) was applied to the blot and the chemiluminescent signals were collected using a CCD camera-based imager, as indicated by the manufacturer. On the ChemiDoc MP imager, image analysis programming was utilized to compare the band intensity of the target protein to the control protein β-actin via protein normalization.

### 2.5 Detection of gasdermin D protein by immunohistochemistry

The mouse polyclonal antihuman GSDMD antibody (catalog no. YPA2109; Chongqing biospes Co. China) was used to stain formalin-fixed paraffin-embedded tissue slides through immunoperoxidase. Four-micron-thick paraffin slides were placed on plus slides and dried for 1 h in a 60°C oven before being deparaffinized on an automated immunostainer Link 48 (DAKO, Denmark) and heat-induced epitope retrieval was conducted for 30 min at 95–100°C with citrate buffer solution, pH 6. At 37°C for 60 min, slides were incubated with the primary antibody, mouse polyclonal anti-GSDMD antibody (1:50 dilution). The (Mouse/Rabbit polydetector DAB HRP Brown detection system Bio SB Santa Barbara CA) was used to visualise immunoreactivity. The slides were dried, cleaned, and mounted using permanent mounting media after being counterstained with hematoxylin. The staining intensity was graded semiquantitatively, with 0 denoting no staining, 1 denoting faint staining, 2 denoting moderate staining, and 3 denoting strong staining (Ramos-Vara and Miller, 2014).

### 2.6 Statistical analysis

IBM-SPSS software (version 25, Armonk, NY: IBM Corp., 2017) and MedCalc® Statistical Software (version 20, MedCalc Software Ltd, Ostend, Belgium) were used to enter and evaluate data. Count (percent) were used to express qualitative data. Quantitative data is mean ± standard deviation as data is normally distributed (Shapiro-Wilk’s test *p* > 0.050). Chi-Square and Fisher’s exact tests were used to compare qualitative data. The Independent-Samples t-test and the one-way ANOVA test were employed to compare quantitative data between two groups and the three research groups, respectively. Significant result in One-Way ANOVA was followed by post-hoc Tukey’s HSD tests and statistical significance was expressed as letters (similar letters = no difference, different letters = significant difference).

Correlation between two continuous variables and between continuous and ordinal variables was assessed by Pearson’s and Spearman’s correlation tests, respectively. The Kaplan-Meier method was used to calculate the survival distribution. The log-rank test was used to determine whether two groups’ survival distributions were equal. The effects of various risk factors on survival were investigated using the Cox proportional-hazards regression model. For simplicity, quantitative predictors were recoded into categorical variables using Receiver Operating Characteristic (ROC) curve analysis. The results were considered statistically significant if the *p* value for any of the tests used was less than 0.050. Appropriate charts were created to graphically portray the data as applicable.

## 3 Results

This study included 80 BC patients divided into two groups based on muscle invasion: **Group 1:** NMIBC (N = 30 patients), and **Group 2:** MIBC (N = 50 patients) Table 1 shows the patient’s and tumor’s characteristics. There was a significant difference in patients′ age; being higher in the MIBC group (mean ± SD of 60.88 ± 0.83 years) versus the NMIBC (mean ± SD of 56.97 ± 1.72 years). This difference was also substantial as shown by large effect size (d = 2.9). There was also a control group of 80 samples from adjacent normal tissue.

**TABLE 1:** Patients and tumor characteristics of the two bladder cancer groups:

Variable	Group		Test of significance	
	NMIBC (n = 30)	MIBC (n = 50)	t/χ^2^	*p* value
Age (years)	56.97 ± 1.72	60.88 ± 0.83	t = -2.049	**0.047**
Sex frequency				
Male	25 (83.3%)	40 (80%)	χ^2^ = 0.137	0.712
Female	5 (16.7%)	10 (20%)		
Cancer grade				
Grade I	0 (0%) _a_	3 (6%) _a_	FET	<0.001*
Grade II	16 (53.3%) _a_	7 (14%) _b_		
Grade III	14 (46.7%) _a_	40 (80%) _b_		
Pathological type				
Transitional cell carcinoma (TCC)	30 (100%) _a_	39 (78%) _b_	FET	0.009*
Adenocarcinoma	0 (0%) _a_	3 (6%) _a_		
Squamous cell carcinoma	0 (0%) _a_	8 (16%) _b_		

Data for age is mean ± SD (test of significance is independent sample t-test), and N (%) for categorical variables (test of significance is Chi-Square (χ^2^) Test or Fisher’s exact test (FET). Bold values indicate significant *p* values (0.050); compact letters show pairwise comparison (similar letters = statistically insignificant difference, while different letters = statistically significant difference); NMIBC = Non-muscle invasive bladder cancer; MIBC = Muscle invasive bladder cancer.

### 3.1 Gadermin D gene expression in the control and bladder cancer tissues

GSDMD gene expression was observed to differ significantly amongst the three groups (One-way ANOVA F = 63.917, *p* < 0.001). When comparing the MIBC group to the NMIBC group, as well as the NMIBC group to the control group, pairwise comparisons revealed a substantial increase in gene expression in the MIBC group (Table 2). This difference was substantial as shown by large effect size (partial η^2^ = 0.449, f = 0.90). There is no statistically significant difference in gene expression across the three pathological types when they are compared. (One-way ANOVA, F = 0.1.710, *p* = 0.188) This difference was also medium as shown by medium effect size (partial η^2^ = 0.043, f = 0.21). (Table 3). Aside from that, there is no significant difference in gene expression between the three tumor grades (One-way ANOVA F = 0.784, *p* = 0.460). This difference was also minor as shown by small effect size (partial η^2^ = 0.020, f = 0.14). (Table 4).

**TABLE 2:** Gasdermin D gene expression (by real-time PCR) and protein expression (by western blotting) in the control tissue samples and samples from cases of the two BC groups:

Variable	Group			Test of significance		
	Control (n = 80)	NMIBC (*n* = 30)	MIBC (*n* = 50)	F	*p* value	Partial η^2^
Gasdermin D gene expression	1.00 ± 0.05	1.32 ± 0.10	2.17 ± 0.11	63.917	**<0.001**	0.449
	A	B	C			
Gasdermin D tissue protein	1.00 ± 0.03	1.34 ± 0.10	1.85 ± 0.10	46.464	**<0.001**	0.372
	A	B	C			

Data are presented as mean ± standard deviation. *p* value by One-Way ANOVA, pairwise comparison using Games-Howell adjustment, post-hoc test is presented as capital letters (Different letters = statistically significant difference), bold values indicate significant *p* values (≤0.050). Partial η^2^ is a measure of effect size.

**TABLE 3:** Gasdermin D gene (by real-time PCR) and protein expression (by western blotting) in the bladder cancer tissue samples from the different pathological types:

Variable	Pathological types			Test of significance		
	TCC (n = 69)	Adenocarcinoma (n = 3)	SCC (n = 8)	F	*p* value	Partial η^2^
Gasdermin D gene expression	1.50 ± 0.08	2.02 ± 0.71	1.86 ± 0.19	1.710	0.188	0.043
Gasdermin D tissue protein	1.30 ± 0.07	1.47 ± 0.46	1.56 ± 0.19	0.895	0.413	0.023

Data are presented as mean ± standard deviation. *p* value by One-Way ANOVA., Partial η^2^ is a measure of effect size.

**TABLE 4:** Gasdermin D gene (by real-time PCR) and protein expression (by western blotting) in the bladder cancer tissue samples from the different pathological grades:

Variable	Pathological grades			Test of significance		
	Grade 1 (*n* = 3)	Grade 2 (*n* = 23)	Grade 3 (*n* = 54)	F	*p* value	Partial η^2^
Gasderim D gene expression	1.57 ± 0.33	1.41 ± 0.15	1.62 ± 0.09	0.784	0.460	0.020
Gasderim D tissue protein	1.22 ± 0.34	1.23 ± 0.11	1.38 ± 0.08	0.599	0.552	0.015

Data are presented as mean ± standard deviation. *p* value by One-Way ANOVA., Partial η^2^ is a measure of effect size.

### 3.2 Gasdermin D protein level in tissue samples

**FIGURE 1:**
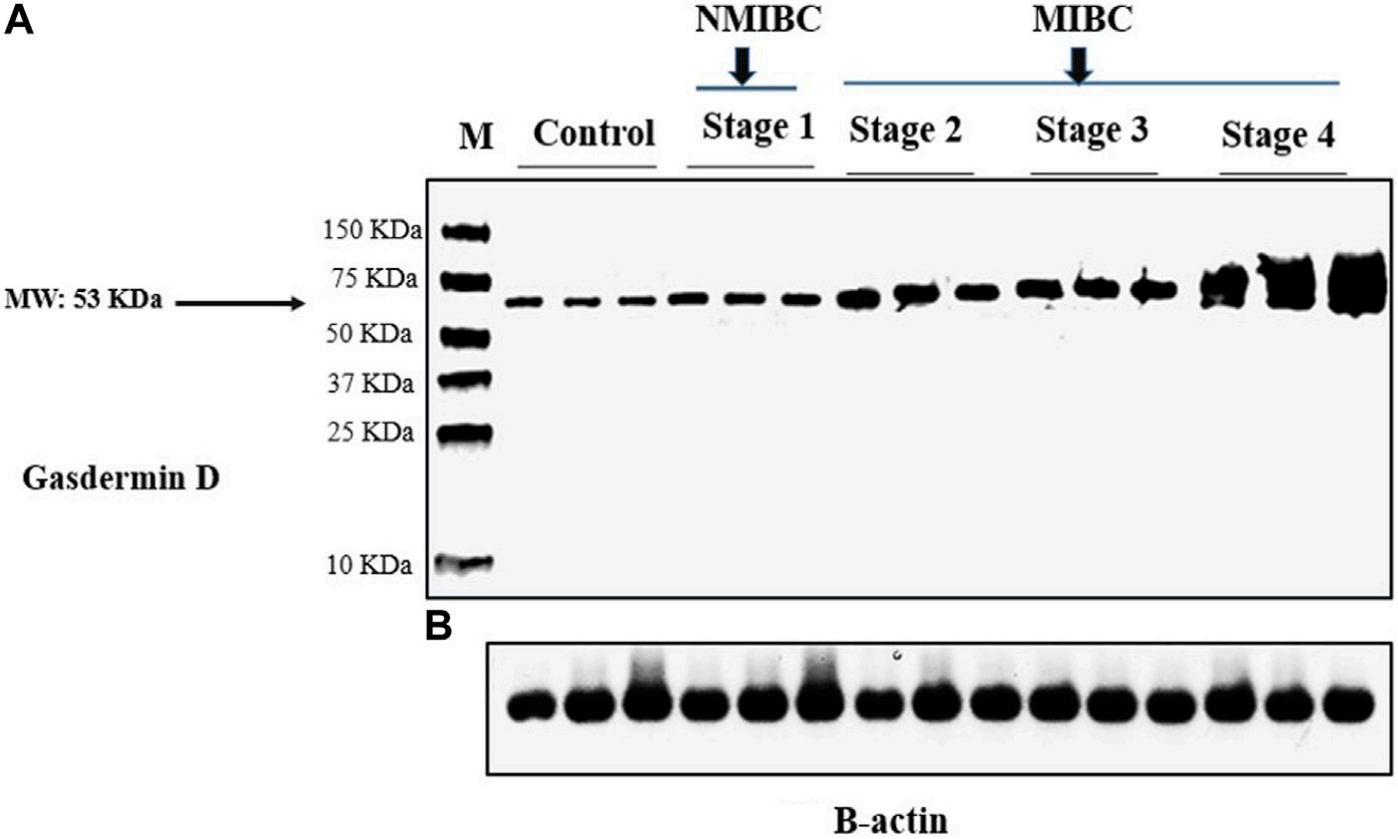
Gasdermin D protein analysis in tissue samples by western blotting in the study groups: **(A)** Gasdermin D protein bands (Molecular weight: 53 kDa). **(B)** β-actin protein bands (Molecular weight: 42 kDa). β-actin was utilized to be an internal control. **NMIBC:** Non-muscle invasive bladder cancer. **MIBC:** Muscle invasive bladder cancer. **M:** Molecular weight marker.

GSDMD protein expression was measured using a Western blot in each of the three groups studied. The difference between the three groups was statistically significant (One-way ANOVA F = 46.464, *p* < 0.001). Pairwise analyses demonstrated higher tissue expression in the MIBC group in comparison to the NMIBC group (Table 2), as well as a significant higher expression in the NMIBC group in comparison to the control (Figure 1). This difference was also substantial as shown by large effect size (partial η^2^ = 0.372, f = 0.77). There is no significant difference in protein expression across the three pathogenic categories (One-way ANOVA F = 0.895, *p* = 0.413), this difference was minor as shown by small effect size (partial η^2^ = 0.023, f = 0.15). (Table 3). Furthermore, when comparing protein expression between the three tumor grades, there is no significant difference (One-way ANOVA F = 0.599, *p* = 0.552), this difference was minor as shown by small effect size (partial η^2^ = 0.015, f = 0.12) (Table 4).

### 3.3 Patients’ characteristics, gasdermin D gene and protein expression in tissue samples in cases with low- and high-grade transitional cell carcinoma (TCC)

The expression of GSDMD gene and protein in cases with low and high grades TCC revealed a statistically significant difference in age and GSDMD gene expression between the lower and upper grades. This difference was also substantial as shown by large effect size (d = 4.14 and 2.11). Although males and females are more in high grade TCC, also the protein expression is higher in high grade TCC than low grade TCC (d = 2.18), there was no statistical significance regarding the sex and protein expression in both groups (*p* = 0.506 and 0.159 respectively) (Table 5).

**TABLE 5:** Patients’ characteristics, gasdermin D gene and protein expression in tissue samples in cases with low- and high-grade transitional cell carcinoma:

Parameter	Group		Test of significance	
	Low grade TCC (*n* = 19)	High-grade TCC (*n* = 51)	t/χ^2^	p
Age (years)				
Mean ± SD	53.95 ± 2.32	61.16 ± 0.82	t = 10.137	**0.008**
Sex				
Male	16 (84.2%)	41 (80.4%)	χ^2^ = 0.133	0.506*
Female	3 (15.8%)	10 (19.6%)		
Gasdermin D gene expression	1.25 ± 0.22	1.61 ± 0.10	t = 2.745	**0.038**
Gasdermin D protein expression	1.15 ± 0.11	1.36 ± 0.08	t = 0.274	0.159

Data are presented as mean ± standard deviation and frequency (percent), *p* value by independent sample t-test and *Chi-square test. Bold values indicate significant *p* values (≤0.050). TCC: Transitional cell carcinoma.

### 3.4 Immunohistochemical Results

**FIGURE 2:**
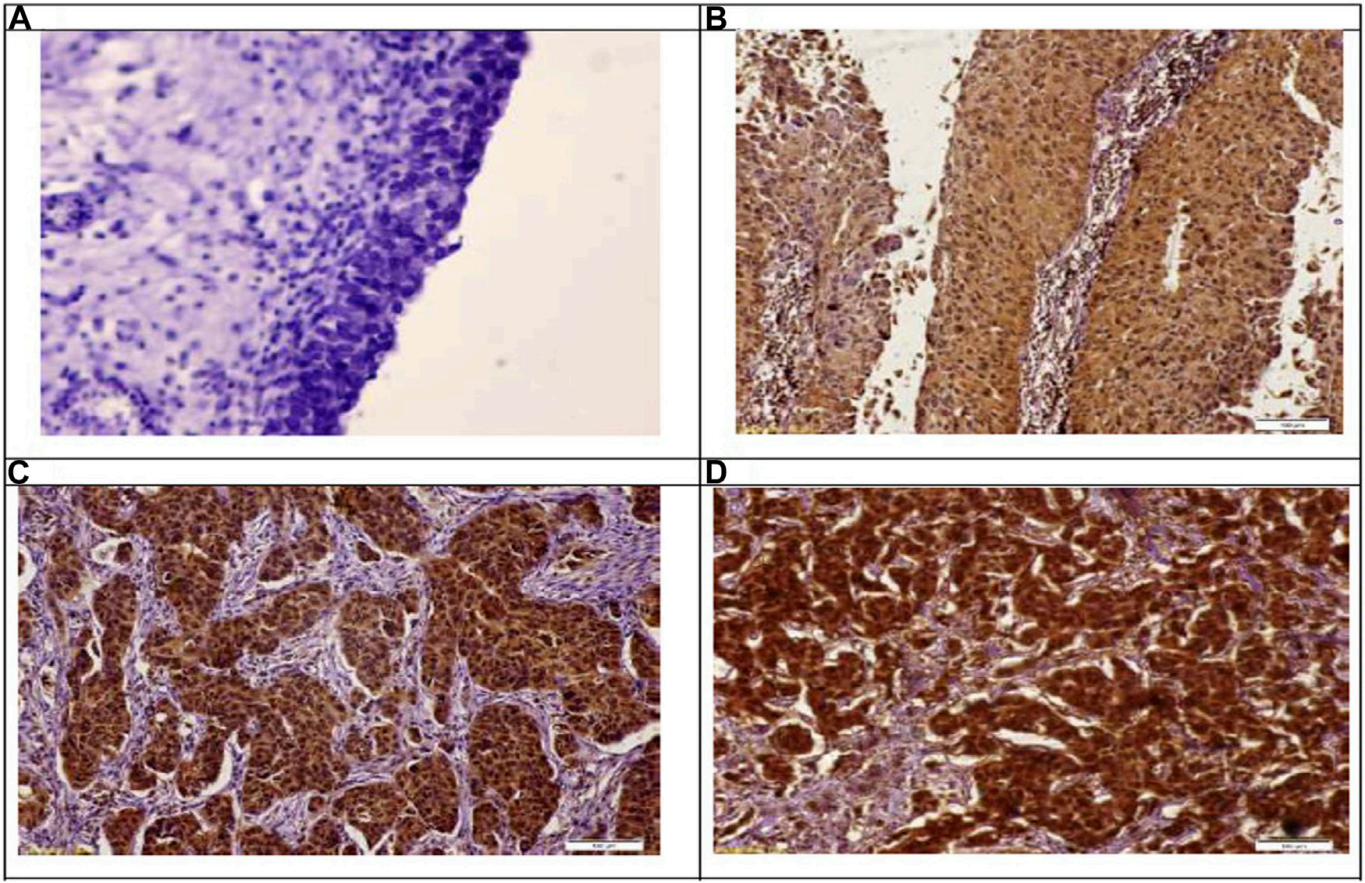
Immunohistochemical staining of gasdermin D in the study groups. **(A):** Showing no expression of gasdermain D at control samples. Immunoperoxidase X 100. **(B):** Showing weak-mild expression of gasdermain D at stage PTa. Immunoperoxidase X 100. **(C):** Showing moderate expression of gasdermain D at stage PT2. Immunoperoxidase X 100. **(D):** Showing marked expression of gasdermain D at stage PT3. Immunoperoxidase X 100.

Immunohistochemical detection of GSDMD protein in the bladder control tissue sections of the negative control group showed a weak immunoreactivity. In contrast, bladder cancer tissue sections of the NMIBC group showed a noticeable cytoplasmic expression of GSDMD, and this expression is increasing with the higher stages showing a strong expression in the MIBC tissue samples. In addition, the inflammatory cells showed the same pattern of GSDMD expression (Table 6) (Figure 2).

**TABLE 6:** Gasdermin D protein expression (by immunohistochemistry) in the bladder cancer tissue samples:

Variable	Group			χ^2^	*p* value
	Control (*n* = 80)	NMIBC (*n* = 30)	MIBC (*n* = 50)		
Gasdermin D					
No/Weak expression	70 (87.5%) _a_	11 (36.7%) _b_	15 (30%) _b_	50.764	**< 0.001**
Moderate/Marked expression	10 (12.5%) _a_	19 (63.3%) _b_	35 (70%) _b_		
Inflammation					
No/Weak inflammation	74 (92.5%) _a_	22 (73.3%) _b_	11 (22%) _c_	69.731	**< 0.001**
Moderate/Marked focal inflammation	6 (7.5%) _a_	8 (26.7%) _b_	39 (78%) _c_		

Data are expressed as count and percent (of total), p value by Chi square test, comparison of column proportions with Bonferroni adjusted p value, bold value indicate significant p values (≤0.050), compact letters demonstrate pairwise comparison (similar letters = statistically insignificant difference, different letters = statistically significant difference). NMIBC = Non-muscle invasive bladder cancer; MIBC = Muscle invasive bladder cancer, χ2= Chi square.

### 3.5 Correlation between gasdermin D gene expression and the other parameters

The correlation between GSDMD gene expression and various clinical, laboratory, and pathological parameters revealed a strong positive correlation with GSDMD protein expression in tissue samples, tumor stage, group, and lymph node involvement, as well as a weak positive correlation with tumor grade. (Table 7).

**TABLE 7:** Correlation between gasdermin D gene expression and the other parameters:

Parameter	Correlation coefficient	*p* value
Gasdermin D protein	0.610	**<0.001**
Age	−0.007	0.953
Largest tumor dimension	0.187	0.097*
Stage	0.534	<0.001*
Grade	0.280	*0.012*
Group	0.627	<0.001*
Lymph node involvement	0.554	<0.001*

*p* value by Pearson′s correlation or *Spearman′s correlation. Bold values indicate significant *p* values (≤0.050).

### 3.6 Survival analysis

**FIGURE 3:**
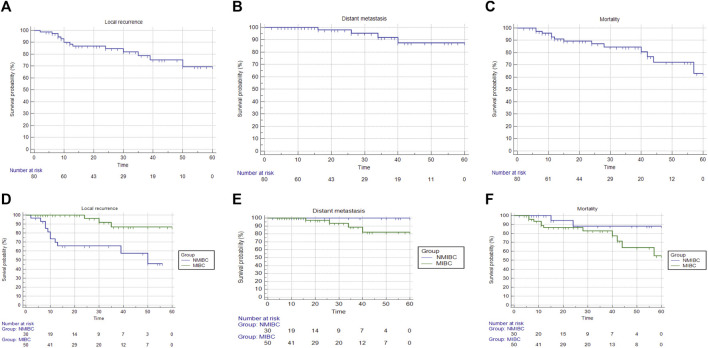
Overall survival and survival analysis of cases in both BC groups. After 5 years, patients were followed up on to see if there was a risk of local recurrence, distant metastases, or mortality. **(A)** Time to local recurrence analysis; overall survival could not be computed since the survival curve did not fall below 0.5. **(B)** Analysis of time to distant metastasis; overall survival could not be computed since the survival curve did not fall to 0.5 or lower. **(C)** Time to death analysis; overall survival could not be computed since the survival curve did not fall below 0.5. **(D)** Analysis of time to local recurrence in both bladder cancer groups; the median survival time for NMIBC was 50 months, but the median survival time for MIBC could not be computed since the survival curve did not drop to 0.5 or lower. **(E)** Analysis of time to distant metastasis; the median survival time for both groups could not be computed. **(F)** Analysis of time to death in both BC groups; the median survival time for both groups could not be computed.

Patients were followed up for a maximum of 60 months period to determine the risk of local recurrence, distant metastasis, and mortality. The median survival which is the smallest time at which the survival probability drops to 0.5 (50%) or below was computed unless the survival curve does not drop to 0.5 or below. In terms of time to local recurrence, the NMIBC group had a median survival time of 60 months, but the MIBC group’s survival curve did not dip below 0.5 or lower, therefore this could not be estimated. The log-rank test was used to compare the survival analyses of the two groups, which revealed a non-statistically significant difference; *p* < 0.001. The median survival time for both groups could not be estimated in terms of time to distant metastases and time to death. A non-statistically significant difference was discovered using the log-rank test; *p* = 0.182 and 0.176, respectively (Table 8) (Figure 3). The restricted mean survival time (RMST) at 36 months was reported (Royston and Parmar, 2013) demonstrating a difference of 3.65 months in time to local recurrence between the two groups (*p* = 0.001), and a difference of 2 months in time to distant metastasis between the two groups (*p* = 0.129), and a difference of 0.61 months in time to death between the two groups (*p* = 0.243).

**TABLE 8:** Survival analysis of cancer patients after 60 months follow up:

Events	Group		Overall survival	p*
	NMIBC (n = 30)	MIBC (n = 50)		
Time to local recurrence				
Number of events	11	3	14	**<0.001**
Censored	19	47	66	
Median survival time	50 months	---	---	
RMST at 36 months	29.73	33.38	32.01	**0.001**
Time to distant metastasis				
Number of events	0	4	4	0.182
Censored	30	46	76	
Median survival time	---	--	--	
RMST at 36 months	33.6	35.6	34.85	0.129
Time to death				
Number of events	2	11	13	0.176
Censored	28	39	67	
Median survival time	--	--	--	
RMST at 36 months	33.23	33.84	33.61	0.243

*p, By log rank test, NMIBC, Non-muscle invasive bladder cancer, MIBC, Muscle invasive bladder cancer, RMST, Restricted mean survival time.

### 3.7 Cox proportional hazards regression models

#### 3.7.1 Time to local recurrence

**FIGURE 4:**
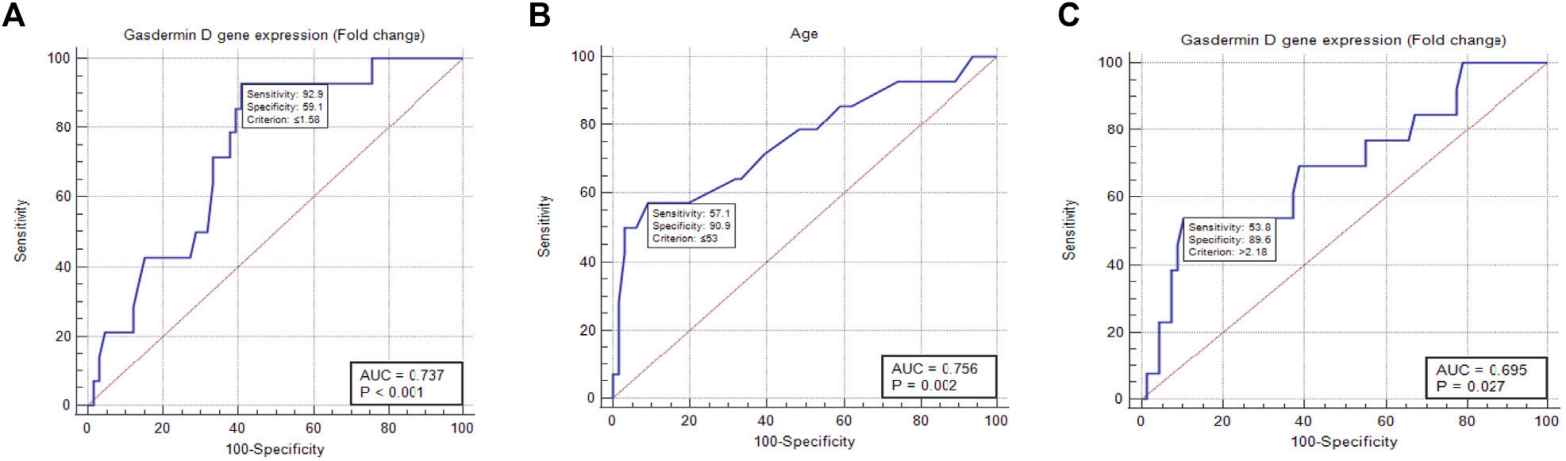
ROC curve analysis of gasdermin D gene expression and age showing the cut-off with best sensitivity and sensitivity to predict the local recurrence and distant metastasis. **(A)** ROC curve showing that gene expression of gasdermin D 1.58-fold is the cutoff to predict local recurrence. **(B)** ROC curve showing that age of 53 years is the cutoff to predict local recurrence. **(C)** ROC curve showing that gene expression of gasdermin D 2.18-fold is the cutoff to predict death.

To determine the effects of GSDMD gene expression and age on the time to local recurrence, a cox model was used. The selection of these predictors was based on testing the factors influencing overall survival as well as each individual predictor separately (Tables 9, 10). Out of the nine predictor variables, only two were statistically significant: GSDMD gene expression (*p* = 0.018, hazard ratio 6.080 and 95% confidence interval of 1.355–27.274), and age (*p* = 0.003, hazard ratio 4.967 and 95% confidence interval of 1.712–14.413) (Table 11). Both predictor variables were recoded into two categories using the ROC curve analysis (Figures 4A,B). Decreased GSDMD gene expression to 1.58-fold or less, and age to 53 years or less were linked to a higher risk of recurrence of a local tumor.

**TABLE 9:** Factors affecting overall survival:

Factor	N	Time to local recurrence			Time to distant metastasis			Time to death		
		Median (95% CI)	Log Rank test		Median (95% CI)	Log Rank test		Median (95% CI)	Log Rank test	
			χ2	P		χ2	P		χ2	P
Age:*										
<60 years	36	44.40 (36.74—52.06)	3.865	**0.049**	54.08 (48.10—60.05)	1.569	0.210	46.62 (39.17—54.07)	2.971	0.085
≥60 years	44	54.52 (49.43—59.62)			58.48 (55.56—61.41)			54.57 (49.50—59.43)		
Sex:										
Male	65	48.89 (43.47—54.32)	0.189	0.664	56.76 (53.29—60.24)	0.085	0.771	49.43 (44.17—54.68)	1.193	0.275
Female	15	52.53 (42.89—62.17)			56.22 (49.24—63.20)			56.40 (49.71—63.09)		
Group:										
NMIBC	30	37.78 (29.33—46.23)	12.186	**<0.001**	-	1.782	0.182	55.23 (49.01 – 61.46)	1.832	0.176
MIBC	50	56.08 (51.92—60.24)			-			48.39 (42.44 – 54.33)		
Grade:										
Grade 1 or 2	26	43.62 (34.89—52.35)	4.140	**0.042**	58.00 (54.28—61.72)	0.315	0.575	54.81 (48.87—60.75)	1.531	0.216
Grade 3	54	53.79 (48.68—58.91)			55.87 (51.46—60.28)			47.72 (41.37—54.08)		
Pathological type:										
TCC	69	48.67 (43.30—54.05)	0.839	0.360	56.80 (53.34—60.26)	0.182	0.670	50.07 (44.94 – 55.21)	0.021	0.884
Adenocarcinoma / SCC	11	55.00 (46.05—63.95)			55.00 (46.51—63.49)			54.18 (45.89 – 62.47)		
Lymph nodes involvement:										
Yes	62	-	3.660	0.056	-	1.109	0.292	53.68 (49.23—58.13)	5.572	**0.018**
No	18	-			-			39.11 (29.04—49.18)		
Largest tumor dimension:*										
< 4 cm	32	47.91 (40.17—55.66)	0.438	0.508	-	2.625	0.105	50.55 (43.21—57.89)	0.022	0.883
≥4 cm	48	50.86 (44.83—56.88)			-			50.69 (44.90—56.48)		
Gasdermin D gene expression:*										
<1.59-fold change	40	41.95 (34.20—49.70)	11.120	**0.001**	58.17 (54.65—61.68)	0.578	0.447	53.75 (48.09—59.40)	1.427	0.232
≥1.59-fold change	40	58.33 (55.18—61.49)			55.35 (50.53—60.17)			48.31 (41.63—54.98)		
Gasdermin D protein expression:*										
<1.27-fold change	40	45.04 (37.27—52.82)	3.683	**0.055**	56.05 (50.85—61.26)	0.099	0.753	49.89 (42.78—57.01)	0.000	0.985
≥1.27-fold change	40	54.15 (48.86—59.45)			56.98 (53.06—60.90)			51.07 (45.04—57.10)		

p: by Log Rank test, Bold values indicate significant p values (≤0.050), NMIBC, Non-muscle invasive bladder cancer, MIBC, Muscle invasive bladder cancer, TCC, Transitional cell carcinoma, SCC: Squamous cell carcinoma, * data converted from being quantitative into categorical based on the median.

**TABLE 10:** Individual predictors of bladder cancer local recurrence, distant metastasis and death:

Predictors	Time to local recurrence		Time to distant metastasis		Time to death	
	P	HR (95% CI)	P	HR (95% CI)	P	HR (95% CI)
Age	**0.002**	0.912 (0.860–0.968)	0.820	0.986 (0.874–1.112)	0.379	0.972 (0.911–1.036)
Sex						
Male	0.666	1.391 (0.311–6.230)	R	R	0.299	2.951 (0.383–22.761)
Female	R	R	0.772	0.714 (0.073–6.958)	R	R
Groups						
NMIBC	**0.003**	7.027 (1.958–25.219)	R	R	R	R
MIBC	R	R	0.431	0.028 (0.000–209.197)	0.195	0.367 (0.081–1.669)
Cancer grade						
Grade 1 or 2	**0.053**	2.967 (0.987–8.913)	R	R	R	R
Grade 3	R	R	0.581	0.527 (0.054–5.131)	0.229	0.451 (0.123–1.651)
Lymph nodes involvement						
Yes	0.237	29.118 (0.109–7793.634)	R	R	R	R
No	R	R	0.516	29.476 (0.001–791,528.922)	**0.027**	0.289 (0.096–0.867)
Largest tumor dimension (cm)	0.469	0.878 (0.617–1.249)	0.258	1.268 (0.840–1.913)	0.063	1.249 (0.988–1.579)
Gasdermin D gene expression	**0.010**	0.240 (0.081–0.713)	0.634	1.440 (0.321–6.469)	**0.054**	2.263 (0.987–5.190)
Gasdermin D tissue protein	**0.039**	0.324 (0.111–0.947)	0.658	0.650 (0.096–4.374)	0.393	1.584 (0.549–4.569)

p: By Cox proportional hazards regression, Bold values indicate significant *p* values (≤0.050), R: Reference group, NMIBC: Non-muscle invasive bladder cancer, MIBC: Muscle invasive bladder cancer, HR: Hazard ratio, **95% CI:** 95% confidence interval.

**TABLE 11:** Cox proportional hazards regression models:

Predictors	Time to local recurrence		Time to death	
	P	HR (95% CI)	P	HR (95% CI)
Gasdermin D gene expression				
≤1.58-fold change	**0.018**	6.080 (1.355–27.274)	**---**	**---**
>1.58-fold change				
Gasdermin D gene expression				
≤2.18-fold change	**---**	**---**	**0.015**	3.924 (0.085–0.766)
>2.18-fold change				
Age				
≤53 years	**0.003**	4.967 (1.712–14.413)	**---**	**---**
>53 years				
Lymph nodes involvement				
Yes	**---**	**---**	**0.046**	3.164 (0.102–0.980)
No				

p: By Cox proportional hazards regression, Bold values indicate significant *p* values (≤0.050), HR, Hazard ratio, 95% CI, 95% confidence interval.

#### 3.7.2 Time to distant metastasis

A cox model could not be performed because the factors affecting overall survival and each individual predictor separately were all non-statistically significant (Tables 9, 10).

#### 3.7.3 Time to death

To determine the effects of GSDMD gene expression and lymph node involvement on the time to death, a cox model was used. The selection of these predictors was based on testing the factors influencing overall survival as well as each individual predictor separately (Tables 9, 10). Out of the nine predictor variables, only two were statistically significant: GSDMD gene expression (*p* = 0.015, hazard ratio 3.924 and 95% confidence interval of 0.085–0.766), and lymph nodes involvement (*p* = 0.046, hazard ratio 3.164 and 95% confidence interval of 0.102–0.980) (Table 11). GSDMD gene expression was recoded into two categories based on the ROC curve (Figure 4C). Increased GSDMD gene expression more than 2.18-fold, and lymph nodes involvement were associated with an increased likelihood of death.

## 4 Discussion

Bladder cancer, usually called as urothelial cancer, is one of the most common cancers in the world (Wigner et al., 2021). Chronic inflammation has a key role in the development of BC. Inflammation can lead to oxidative stress and the generation of proinflammatory cytokines including interleukins (ILs) and tumour necrosis factor (TNF-α), which are both involved in carcinogenesis (Reuter et al., 2010). Pyroptosis is a pro-inflammatory type of apoptosis. The role of pyroptosis mediators in cancer pathogenesis is not fully understood (Fanga et al., 2020).

Gasdermins have been identified as a key mediator of pyroptosis. This family also includes GSDMA, GSDMB, GSDMC, DFNA5, and DFNB59. GSDMA and GSDMB are both found on human chromosome 17q21.1, but GSDMC and GSDMD are found on chromosome 8q24 (Qiu et al., 2017). The gasdermin family is 45 percent homologous in terms of overall sequence. Gasdermin structures are made up of two domains: gasdermin-N and -C domains (Van Rossom et al., 2012). Pyroptosis is caused by the gasdermin-N domains disrupting the cellular membrane (Qiu et al., 2017).

According to the findings of this study, The MIBC group had considerably higher GSDMD gene expression than the other groups which is in agreement with Peng et al. (2020). Although this study followed the patients for a shorter period of time, the results were consistent with the current study.

The current study recorded to the best of knowledge a substantial difference in GSDMD protein levels in tissue samples between the BC and control groups, which correlates with GSDMD gene expression data. There was no evidence of link between the GSDMD gene and tissue protein and the grade or histologic cell type of the tumor. These findings are in agreement with those of Gao et al. (2018) who observed that GSDMD expression was highly elevated in non-small cell lung cancer (NSCLC) (Gao et al., 2018). Through the EGFR/AKT signalling pathway, GSDMD increases lung cancer proliferation as well as an unfavorable prognosis. In contrast, these findings contradict Kayagaki et al. (2015) who observed a significant reduction in GSDMD expression in gastric cancer cells when compared to normal gastric cells. This disagreement may be due to forced GSDMD overexpression, which can reverse gastric cancer cell proliferation by inactivating the ERK, STAT3, and PI3K/AKT pathways, and then inhibiting the Cyclin A2/CDK-2 complex to arrest the S/G2 phase transition (Wang et al., 2018). Wang et al. (2021) stated that GSDMD may play a dual role in carcinogenesis; GSDMD can be broken in the central linker region by caspase-1/4/11, resulting in a GSDMD N-terminal fragment (NT) and a GSDMD C-terminal fragment (NT) (CT). GSDMD-NT is a transmembrane protein that helps cells and organelles create transmembrane holes (such as mitochondrial or nuclear membrane). Substance exchange and pyroptosis are caused by the presence of GSDMD-NT holes in the cellular membrane. Organelle membrane GSDMD-NT holes are also involved in pyroptosis and other processes. GSDMD-NT, for example, stimulates the generation of reactive oxygen species when it targets the mitochondrial membrane, as well as a systemic inflammatory response and tumour immune microenvironment (Wang et al., 2021).

Other studies have presented the theory of pyroptosis’ dual processes. Pyoptosis may inhibit the onset of carcinogenesis on the one hand, but as a sort of proinflammatory death, it may also produce an ideal microenvironment for cancer cell growth, implying that it has both stimulating and inhibitory functions (Xia et al., 2019).

In the current study, cox proportional hazards regression models revealed that GSDMD gene expression was 1.58-fold higher in cancer patients, and age 53 years was associated with a higher likelihood of local tumour recurrence. Furthermore, increased GSDMD gene expression >2.18-fold and lymph node involvement were linked to an increased risk of death. These findings correspond to the findings of Xia et al. (2021) who discovered a favorable link between high expression of GSDMD and prognosis and therapeutic response in breast cancer, and GSDMD is a promising prognostic diagnostic and predictor of treatment efficacy in invasive breast cancer (Xia et al., 2021).

Wu et al. (2020) found the link between GSDMD high expression and worse outcome in colorectal cancer (CRC) (Wu et al., 2020). Other studies, however, have found that levels of essential pyroptosis cascade molecules such NLRP1, NLRP3, and AIM2 are lower in CRC with a poor outcome (Markowitz et al., 2016; Zhou and Fang, 2019). Furthermore, high GSDMD expression has been linked to longer overall survival and less cancer cell invasion in breast cancer. However, in adenoid cystic carcinoma (ACC), GSDMD increased ACC cell invasiveness, indicating that high GSDMD expression was associated with a poor prognosis (Shen et al., 2020). According to previous studies, the lncRNA RP1-85F18.6 can cause cell pyroptosis in CRC by activating GSDMD, which has some prognostic value (Ruan et al., 2020).

There are few studies on the role of GSDMD as s promising prognostic marker in BC, hence additional research is needed to back up the conclusions. Our research could aid in the early prediction of BC outcome.

## 5 Summary and conclusion

The current research revealed higher GSDMD gene and protein expression in MIBC in comparison with the normal and NMIBC groups. In addition, GSDMD gene expression in cancer patients is a positive predictor of local tumor recurrence. Moreover, increased GSDMD gene expression, and lymph nodes involvement are associated with an increased likelihood of death.

## 6 Recommendations

Gasdermin D should be tested on a bigger scale with a longer follow-up period to confirm its value as a prognostic marker for bladder cancer. Also, we recommend researchers to attempt combination of pyroptosis regulation together with inhibiting the proliferation, migration and invasion of tumor cells as therapeutic targets for bladder cancer.

## Data Availability

The original contributions presented in the study are included in the article/supplementary materials, further inquiries can be directed to the corresponding author.
